# Paeoniflorin ameliorates glycemic variability-induced oxidative stress and platelet activation in HUVECs and DM rats

**DOI:** 10.1039/d0ra02036b

**Published:** 2020-11-24

**Authors:** Ye Huang, Jing-Shang Wang, Lin Yang, Long Yue, Lei Zhang, Yan-Hong Zhang, Ye-Wen Song, Dandan Li, Zhixu Yang

**Affiliations:** Emergency Department, Xiyuan Hospital, China Academy of Chinese Medical Sciences Beijing 100091 China yangzhixu@sohu.com +86 10 62835314 +86 10 62835314; Department of Traditional Chinese Medicine, Beijing Obstetrics and Gynecology Hospital, Capital Medical University Beijing 100026 China; Department of Cardiovascular Disease, Xiyuan Hospital, China Academy of Chinese Medical Sciences Beijing 100091 China; Institute of Geriatrics, Xiyuan Hospital, China Academy of Chinese Medical Sciences Beijing 100091 China

## Abstract

Glycemic variability (GV) plays an important role in the pathogenesis of vascular complications associated with diabetes mellitus (DM). Paeoniflorin is an effective Chinese traditional medicine with anti-inflammatory and immune-regulatory effects. Previous studies implicated the beneficial effects of paeoniflorin in treatment for diabetic complications, such as type 2 diabetic nephropathy and diabetes with myocardial ischemic injury. Current evidence suggests that oxidative stress and platelet activation, as well as their interaction, are potentially associated with GV and involved in the pathogenesis of diabetes-associated vascular complications. This study aimed to explore the effects of paeoniflorin on oxidative stress and platelet activation, using human umbilical vein endothelial cells (HUVECs) cultured with different glucose concentrations, and streptozotocin-induced diabetic rats fed different glycemic index diets. Paeoniflorin treatment effectively improved the morphology and cell viability of HUVECs under glucose fluctuation. Moreover, the platelet aggregation rate, CD62p expression, and reactive oxygen species (ROS) concentration decreased, while glutathione peroxidase (GSH-px) levels increased in paeoniflorin-treated groups. In conclusion, our study found that paeoniflorin ameliorates oxidative stress and platelet activation induced by glycemic variability both *in vivo* and *in vitro*, suggesting a novel potential strategy for treatment of diabetic complications.

## Abstract 2

Glycemic variability (GV) plays an important role in the pathogenesis of vascular complications associated with diabetes mellitus (DM).

## Graphical abstract

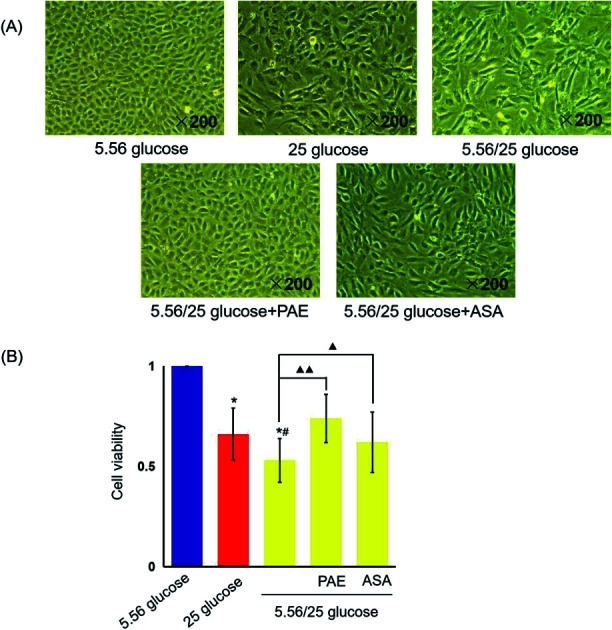

## Introduction

Diabetes mellitus (DM) is a global health issue associated with substantial negative outcomes. Macro- and microvascular complications are the main cause of morbidity and mortality in diabetic patients.^1^ Type 2 diabetes mellitus (T2DM) is the most prevalent form of diabetes, accounting for approximately 90–95% of total diabetes cases worldwide. Glycemic control is essential in the management of patients with T2DM to prevent cardiovascular disease and related complications.^2^ Notably, both *in vivo* and *in vitro* evidence suggests that increased glucose variability causes more severe endothelial damage compared to prolonged hyperglycemia.^3^ Glycemic variability (GV) could, therefore, be an independent risk factor for development of diabetic complications.^4^ Additional evidence points to a potential link between GV and development of complications such as diabetic peripheral neuropathy,^5^ cardiovascular autonomic neuropathy,^6^ and stroke.^7^

Endothelial dysfunction is the key event that initiates the inflammatory mechanisms associated with vascular complications in patients with T2DM.^8^ Activation of the endothelium by increased release of cytokines and expression of adhesion molecules mediates platelet activation and adhesion to the activated endothelium.^9^ Platelets contribute to T2DM-associated cardiovascular complications by not only triggering thrombus formation but also releasing oxidative, mitogenic, and vasoconstrictive substances that induce the development of local vascular lesions.^10^ Diabetes is associated with systemic inflammation and oxidative stress, which may contribute to increased platelet reactivity.^11^ Abnormal platelet function has been identified as one of the mechanisms of enhanced arterial thrombosis in T2DM.^12^ P-selectin, also known as CD62p, is a cell adhesion receptor playing a key role in hemostasis. P-selectin mediates the adhesion of activated platelets to neutrophils and monocytes, thereby facilitating the innate immune response, as well as inducing platelet-to-platelet binding and aggregation.^13^ Consequently, platelet activation induces an increase in soluble P-selectin (sP-selectin),^14^ which can therefore be used as a surrogate marker of this event. Moreover, oxidative stress is also associated with the incidence of diabetes and plays a key role in its complications.^15^

Paeoniflorin (PAE), an ingredient of a typical Chinese herbal medicine, is a bioactive component of total glucosides of paeony, extracted from the dried peeled root of *Paeonia lactiflora* Pall.^16^ PAE exerts numerous pharmacological effects, and has been widely used as an anti-inflammatory, anti-tumor, and immunomodulatory agent.^17–19^ Several studies highlight the beneficial effects of PAE in rat and mouse models of diabetes. PAE plays a protective role in mice with diabetic nephropathy by inhibiting the JAK2/STAT3 signaling pathway.^20^ It also relieves diabetes-associated cognitive deficits in diabetic rats *via* regulating the SOCS2/IRS-1 pathway.^21^ Finally, PAE protects diabetic mice against myocardial infarction, at least partially, *via* the TRPV1/CaMK/CREB/CGRP signaling pathway.^22^ However, the effects of PAE on platelet activation and oxidative stress in the context of GV have not been investigated yet. Therefore, in this study, human umbilical vein endothelial cells (HUVECs) cultured with different glucose concentrations and streptozotocin (STZ)-induced diabetic rats fed with different glycemic index diets were used to examine these effects. Moreover, we determined the effects of PAE on platelet activation and oxidative stress induced by GV.

## Experimental

### Culture and treatment of HUVECs

HUVECs were isolated and pooled from healthy puerperal human umbilical cords after obtaining the appropriate informed consent, following the procedure described by Jaffe *et al.*^23^ HUVECs were cultured under different GV conditions for 8 days as previously described.^24^ Briefly, normal glucose medium (5.56 mmol L^−1^), sustained high-glucose medium (25 mmol L^−1^), or fluctuant high-glucose medium (5.56/25 mmol L^−1^) in which normal and high-glucose media were alternated every 24 h, were applied with daily PAE (100 μM, Dalian Meilun Biotechnology, China) or aspirin (1 mM, Bayer Medical Care Co. LTD, China) treatment.

### Cell morphology and viability

HUVECs were seeded at a density of 5 × 10^3^ cells per well in 96-well plates. After different treatments for 8 days, morphological changes and viability of HUVECs were observed by inverted light microscopy (DMIRB, Leica, Germany) and Cell Counting Kit-8 (CCK-8), according to manufacturer's instructions (Yeasen Biotech Co., Ltd, China.) Briefly, HUVECs seeded at a density of 5 × 10^3^ cells per mL were incubated at 37 °C for 24 h, after which 10 μL mL^−1^ of CCK-8 regent was added to each well, and cells were incubated at 37 °C for a further 4 h. Cell viability was measured at 450 nm on a spectrophotometer (Eppendorf, Germany). The ratio of absorbance of each group to that of the HUVECs cultured with normal glucose medium was used to evaluate cell viability.

### Human platelets and platelet aggregation rate

Blood samples from healthy adult volunteers (*n* = 15, aged 20–45 years) with no history of atherothrombosis, diabetes, or use of medication known to affect platelet function within the past 2 weeks, were collected by antecubital venipuncture into 3.8% sodium citrate vacuum tubes (Greiner bio-one, Austria) containing 1 volume citrate per 9 volumes of blood, according to previous protocols.^25^ Informed consent was obtained from all subjects and the study was approved by Ethics Committee of Xiyuan Hospital of China Academy of Chinese Medical Sciences. Procedures were performed in accordance with the ethical standards of the responsible committee on human experimentation (institutional and national) and with the Helsinki Declaration of 1975, as revised in 2008. The whole-blood specimen was centrifuged for 10 min at 200 × *g* to obtain platelet-rich plasma (PRP). Platelet-poor plasma (PPP) was obtained from the remaining specimen *via* re-centrifugation at 765 × *g* for 10 min. A platelet count was performed on the PRP and was adjusted to 200–300 × 10^9^/L with PPP. Three hundred microliters of this PRP was transferred into cuvettes containing one disposable siliconized bar. Following agonist addition, platelet aggregation was measured over 6 min and expressed as a percentage of the maximal amplitude in PRP.

For platelet aggregation rate, HUVECs cultured in normal glucose, sustained high-glucose medium, or fluctuant high-glucose medium for 8 days were pretreated either with distilled water, PAE, or ASA for 15 min, and then incubated with 300 μL PRP of healthy donors. The culture supernatants were collected, and the platelet aggregation rate was measured by the Born method as described.^26^ The maximum aggregation rate was monitored with a platelet aggregator (LBY-NJ2, Beijing Pulisheng Instrument Co., LTD. China) (Fig. 5A).

### Animals and treatments

Male Sprague Dawley (SD) rats (6 weeks old, weighing 180 ± 10 g), were purchased from the National Institute for Food and Drug Control (SCXK 2009-0017). Rats were housed under a standard specific pathogen-free (SPF) environment for 14 days, with water and food *ad libitum* provided by Beijing Huafukang feed company. All animal experiments were conducted in accordance with the Guidelines for the Care and Use of Laboratory Animals of North China University of Science and Technology, and were approved by the Animal Ethics Committee of North China University of Science and Technology.

Rats were fed with a high-glucose and high-fat diet (containing 68.5% basal diet, 20% lard, 10% sucrose, 1.25% cholesterol, 0.25% cholate, Beijing Nuokangyuan Bio-Technique Co. Ltd) for 4 weeks, to induce insulin resistance. After the 4^th^ week, T2DM was induced according to the method of Srinivasan *et al.*^27^ by intraperitoneal injection of a low dose of STZ (30 mg kg^−1^) dissolved in freshly prepared citrate buffer. Three days after induction, rats with fasting blood glucose (FBG) levels > 11.1 mmol L^−1^ and blood glucose concentration 2 h postprandium > 33.3 mmol L^−1^, measured in tail vein blood samples, were considered diabetic and selected for our study.

T2DM rats were randomly divided into the following groups. Rats in the normal control (NC, *n* = 10) group were fed with a normal diet. Rats in the diabetes with sustained hyperglycemia (DSH, *n* = 11) group were fed with low-glycemic index (GI) forage. Rats in the diabetes with fluctuant hyperglycemia (DFH, *n* = 11) group were fed with high-GI forage. Rats in the diabetes with fluctuant hyperglycemia + PAE (*n* = 10) group were fed with high-GI forage and given 10 mg kg^−1^ d^−1^ PAE (LOT: 20121015, Dalian Meilun Biotechnology Co. Ltd) by intragastric administration once a day for 6 weeks. Rats in the diabetes with fluctuant hyperglycemia + aspirin (*n* = 10) group were fed with high-GI forage and given 60 mg kg^−1^ d^−1^ aspirin (LOT: BJ10194, Bayer Medical Care Co. Ltd) by intragastric administration once a day for 6 weeks. The NC, DSH, and DFH groups all received the same volume of physiological saline by intragastric administration once a day for 6 weeks. Detailed experimental protocols are shown in Fig. 5B. The establishment and identification of rats with different GVs were previously described.^34^

### Platelet glycoprotein CD62p expression

The expression of P-selectin was determined in peripheral blood platelets from rats using flow cytometry (FACS) analysis according to the procedures described previously.^28^ Monoclonal antibodies to P-selectin were labeled with phycoerythrin and those specific for glycoprotein IIIa (CD61) were labeled with fluorescein isothiocyanate. Both antibodies were directly labeled and purchased from Becton Dickinson.

### Peroxidation and antioxidant assays

The cell culture supernatant was collected in a 1.5 mL sterile centrifuge tube and centrifuged at 800 × *g* for 20 min at 37 °C. Blood was collected from the abdominal aorta in rats and serum was obtained by centrifuging at 1000 × *g* for 15 min at 37 °C after anesthetization. The concentration of ROS and GSH-px protein was quantified in cell supernatants from HUVECs, or serum from rats using ELISA kits (Westang Biotech, Shanghai, China), according to the manufacturer's protocol.

### Statistical analysis

The data are presented as means ± standard deviation (SD) and were analyzed by SPSS software version 16.0 (Inc., Chicago, IL, USA). The normality was assessed using the Shapiro-Wilk test. One-way ANOVA was used to analyze multiple comparison tests. The difference between groups was tested by least significant difference (LSD), and Levene method was used for homogeneity testing of variance. *P* values < 0.05 were considered significant.

## Results

### PAE restores cell morphology and cell viability in HUVECs exposed to GV

**Fig. 1:**
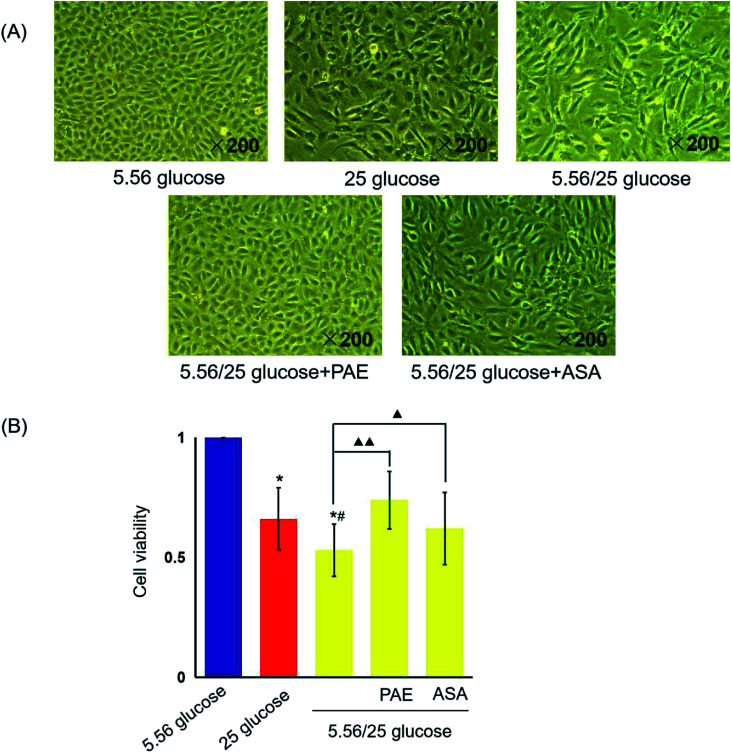
Effects of paeoniflorin (PAE) on the morphology and viability of human umbilical vein endothelial cells (HUVECs) cultured with different concentrations of glucose. HUVECs were cultured in the presence of normal (5.56 mmol L^−1^), sustained high glucose (25 mmol L^−1^), or fluctuant high glucose (5.56/25 mmol L^−1^) for 8 days and treated with PAE or ASA. (A) Morphology of cells treated as described above, observed using an inverted light microscope. (B) Viability of treated cells was measured using CCK-8 kit. Data are presented as the mean ± standard deviation (*n* = 6). *P < 0.01 *vs.* glucose 5.56 mmol L^−1^; ^#^*P* < 0.05 *vs.* glucose 25 mmol L^−1^; ^▲^*P* < 0.05, ^▲▲^*P* < 0.01 *vs.* glucose 5.56/25 mmol L^−1^.

**Fig. 2:**
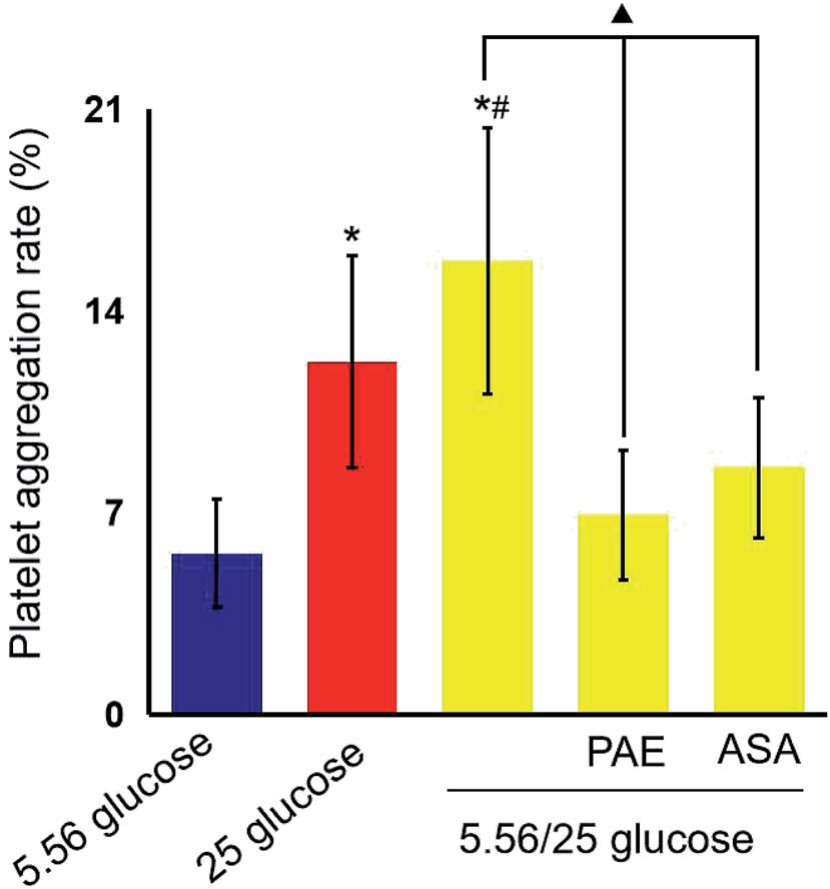
Effects of paeoniflorin (PAE) on platelet aggregation rate. HUVECs were cultured in the presence of normal glucose (5.56 mmol L^−1^), sustained high glucose (25 mmol L^−1^), or fluctuant high glucose (5.56/25 mmol L^−1^). Cells were pre-treated with PAE or ASA for 15 min and then incubated with platelet-rich plasma (PRP) from 15 healthy donors. Fifteen data were obtained in each group. Data are presented as mean ± standard deviation. *P < 0.01 *vs.* glucose 5.56 mmol L^−1^; ^#^*P* < 0.01 *vs.* glucose 25 mmol L^−1^; ^▲^*P* < 0.01 *vs.* glucose 5.56/25 mmol L^−1^.

HUVECs cultured with normal glucose concentrations were adherent and arranged evenly like pebbles (Fig. 1A). Cells had distinct borders, abundant cytoplasm, and an elliptic nucleus. However, many adherent HUVECs grown in sustained high-glucose and fluctuant glucose medium were fused and showed enlarged intercellular gap and swelled cytoplasm. More serious morphological damages were observed in HUVECs grown with fluctuant glucose than in those grown with sustained high glucose. However, the morphology of cells cultured with fluctuant glucose clearly improved upon treatment with PAE and ASA, with a more pronounced improvement in PAE-treated cells (Fig. 2A).

Sustained high glucose and fluctuant glucose reduced the viability of cells by 34% and 47%, respectively (*P* < 0.01, Fig. 1B). However, viability of cells cultured with fluctuant glucose significantly increased upon PAE (40%, *P* < 0.01) and ASA (17%, *P* < 0.05) treatment.

### PAE inhibits platelet aggregation in HUVECs exposed to GV and healthy platelets

Next, platelets from healthy donors were incubated with HUVECs under different GV treatments. As shown in Fig. 2, HUVECs cultured in sustained high-glucose and intermittent high-glucose medium showed a higher maximum platelet aggregation rate than those cultured with normal glucose (*P* < 0.01). Moreover, the difference between HUVECs cultured with sustained high glucose and fluctuant glucose was significant (*P* < 0.01). Importantly, PAE and ASA treatment decreased the maximum platelet aggregation induced by intermittent high glucose (*P* < 0.01). Although the PAE group displayed a higher rate of maximum platelet aggregation than the ASA group, the difference was not significant.

### PAE improves peroxidation and antioxidant activities in HUVECs and T2DM rats exposed to GV

**Fig. 3:**
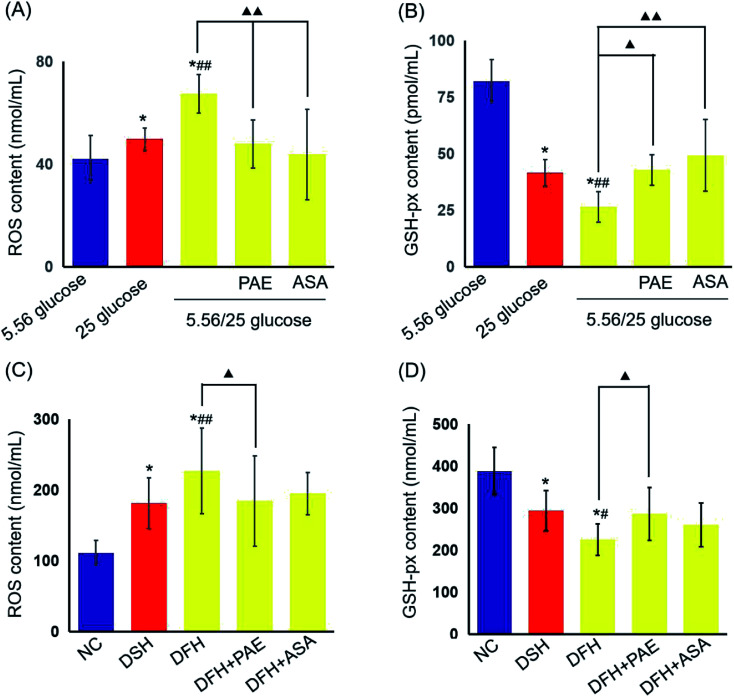
Effects of PAE on expression of reactive oxygen species (ROS) and GPx in HUVECs cultured with different concentrations of glucose and T2DM rats fed with different glycemic index (GI) diets. The levels of ROS (A) and GSH-px (B) in HUVEC supernatant. The levels of ROS (C) and GSH-px (D) in sera. There are 6 data in each group *in vitro* experiment. There are 11 rats in both DSH group and DFH group, and 10 rats in the other group. Date are presented as mean ± standard deviation. *P < 0.01, compared with HUVECs cultured in 5.56 mmol L^−1^ glucose or the NC group. ^#^*P* < 0.05, ^##^*P* < 0.01, compared with HUVECs cultured in 25 mmol L^−1^ glucose or the DSH group. ^▲^*P* < 0.05, ^▲▲^*P* < 0.01, compared with 5.56/25 mmol L^−1^ glucose or the DFH group.

We then evaluated the levels of reactive oxygen species (ROS) and glutathione peroxidase (GSH-px) in HUVECs (Fig. 3A and B) and T2DM rats (Fig. 3C and D). Sustained high glucose and fluctuant glucose increased the levels of ROS in HUVECs by 15% and 37%, respectively (Fig. 3A) and decreased the levels of GSH-px by 50% and 68%, respectively (Fig. 3B). In both cases, intermittent high glucose induced a greater change than sustained high glucose. Importantly, treatment with both PAE and ASA inhibited the intermittent high glucose-induced increase in ROS levels and decrease in expression of GSH-px (*P* < 0.01). Furthermore, an evaluation of ROS and GSH-px levels in the serum of T2DM rats demonstrated similar results (Fig. 3C and D), consistent with the *in vitro* observations (Fig. 3A and B).

### PAE blocks the increase in platelet glycoprotein CD62p induced in T2DM rats by GV

**Fig. 4:**
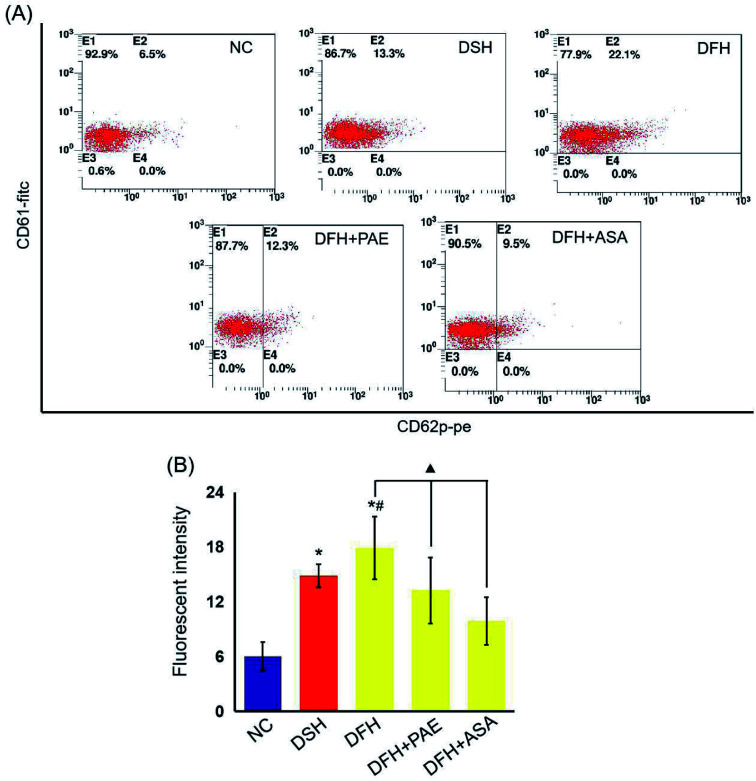
Effects of PAE on platelet activation in T2DM rats fed with different GI diets. (A) Representative data of FACS analysis of platelet glycoprotein CD62p. Whole peripheral-blood samples from experimental rats were stained with PE-conjugated anti-CD62p antibody and FITC-conjugated anti-CD61 antibody, followed by FACS. (B) The CD62p levels was reflected by its fluorescence intensity. There are 11 rats in both DSH group and DFH group, and 10 rats in the other groups. Data are presented as mean ± standard deviation. *P < 0.01, compared with the NC group. ^#^*P* < 0.05, compared with the DSH group. ^▲^*P* < 0.01, compared with the DFH group.

As shown in Fig. 4, the expression of platelet glycoprotein CD62p in T2DM rats was increased in both DSH and DFH groups (*P* < 0.01), and the difference between these two groups was significant (*P* < 0.05). Moreover, pretreatment with both PAE and ASA blocked the increase in platelet glycoprotein CD62p (Fig. 4, *P* < 0.01) in the DFH group (Fig. 4, *P* < 0.01).

**Fig. 5:**
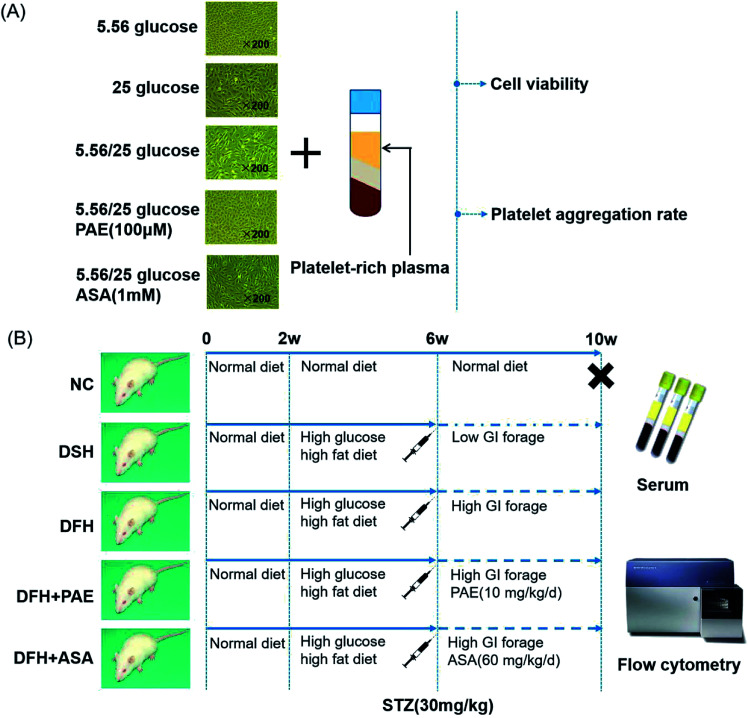
Workflow of the study. (A) Human umbilical vein endothelial cells (HUVECs), platelet-rich plasma of healthy donors, and treatment procedure. (B) Animals and treatment procedure.

## Discussion

Diabetes is a major cause of morbidity and mortality worldwide. Patients with DM are at a high risk of adverse micro- and macrovascular complications. DM-related complications depend primarily on glycemic disorders, which have two main components: long-term chronic hyperglycemia and acute glycemic fluctuations between high and low values (GV).^29^ Recent studies have revealed that not only constant hyperglycemia, but also large GV, may influence cardiovascular risk in DM patients, and that GV has even more deleterious effects than sustained hyperglycemia.^30^ Moreover, GV was suggested as an independent risk factor for development of diabetic complications.^4^

Furthermore, accumulating evidence implicates endothelial dysfunction and damage as early stages in the pathophysiology of vascular complications in DM.^31^ Our previous study reported induced apoptosis following treatment of HUVECs with high glucose concentrations.^24^ Thus, HUVECs was selected as model cell line in our current study. We found that both stable high glucose and intermittent high glucose (corresponding to GV) exposure decreased the viability, while increasing the damage caused to HUVEC morphology, which was consistent with results from a previous study.^32^

PAE, a component of a traditional Chinese medicine, has multiple reported biological properties including, anti-inflammatory, anti-oxidative, and anti-apoptotic, and has, therefore, been applied to the treatment of various cardio-cerebral vascular diseases.^33^ Our previous study revealed that PAE could attenuate vascular injuries induced by fluctuant hyperglycemia through oxidative stress inhibition, inflammatory reaction reduction, and PKCβ1 protein level repression.^24^ Hyperglycemia can increase platelet reactivation *via* numerous mechanisms, including glycation of platelet surface proteins, which increases platelet adhesion by impairing membrane fluidity.^34^ Hyperglycemia-induced oxidative stress is also an important biochemical link between impaired glycemic control and persistent platelet activation.^35^ However, to date, the effect of PAE on platelet status induced by hyperglycemia has not been reported.

P-selectin is a carbohydrate-binding lectin stored in platelets, as well as in endothelial cells, and is well known as a marker of platelet activation.^36^ Circulating levels of sP-selectin have been proposed as a biomarker of macrovascular and microvascular dysfunction in DM.^37^ In this study, we used aspirin as a positive control. Aspirin is a classic antiplatelet drug that reduces the risk of cardiovascular events in patients with diabetes through the irreversible inhibition of cyclooxygenase (COX)-1, a key enzyme involved in vascular clot formation.^38^ In our study, we found increased platelet aggregation rate and high level of CD62p under GV *in vitro* and *in vivo*, respectively. Notably, the ability of PAE to alleviate platelet hyperaggregability induced by GV was similar to that of aspirin.

Oxidative stress due to overproduction of ROS plays a key role in the induction and progression of diabetic complications, both microvascular and cardiovascular.^39^ Glucose fluctuations can induce excessive formation of ROS more than sustained chronic hyperglycemia, ultimately leading to oxidative stress-related apoptosis.^40^ Glutathione (GSH, or γ-l-glutamyl-l-cysteinylglycine) is an important non-enzymatic cellular antioxidant and is a cofactor of GPx. Reduced GSH concentration was found in patients with T2DM.^41^ Moreover, the overproduction of reactive oxygen and nitrogen species, as well as that of potent radicals in patients with DM is the direct cause of increased platelet activation.^42^ In our study, ROS concentration was increased and expression of GSH-px decreased in culture supernatants of HUVECs grown under GV conditions and in sera of T2DM rats fed different GI diets. In fact, the inhibitory effect of PAE on oxidative stress was similar to that of aspirin.

## Conclusions

In conclusion, our study confirmed that PAE could improve cell viability, inhibit platelet activation, and alleviate oxidative stress in GV-exposed HUVECs, as well as reduce oxidative stress and overexpression of CD62p in GV-exposed T2DM rats. As described, endothelial cell injury induced by platelet activation and oxidative stress participates in the development and progression of diabetes-related vascular complications. Interactions between platelet hyperaggregation and oxidative stress in diabetes and the effect of PAE on these mechanisms need further clarification.

## Abbreviations

ASA: Aspirin (acetylsalicylic acid)
CD62p: P-selectin
CVD: Cardiovascular disease
DFH: Diabetes with fluctuant hyperglycemia
DM: Diabetes mellitus
DSH: Diabetes with sustained hyperglycemia
GSH-px: Antioxidant enzymes and glutathione peroxidase
GV: Glycemic variability
HUVECs: Human umbilical vein endothelial cells
PAE: Paeoniflorin
PPP: Platelet-poor plasma
PRP: Platelet-rich plasma
ROS: Reactive oxygen species
T2DM: Type 2 diabetes mellitus

## Conflicts of interest

There are no conflicts to declare.

## Supplementary Material
